# TGF-β Small Molecule Inhibitor SB431542 Reduces Rotator Cuff Muscle Fibrosis and Fatty Infiltration By Promoting Fibro/Adipogenic Progenitor Apoptosis

**DOI:** 10.1371/journal.pone.0155486

**Published:** 2016-05-17

**Authors:** Michael R. Davies, Xuhui Liu, Lawrence Lee, Dominique Laron, Anne Y. Ning, Hubert T. Kim, Brian T. Feeley

**Affiliations:** 1 Department of Veterans Affairs, San Francisco Veterans Affairs Medical Center, San Francisco, California, United States of America; 2 Department of Orthopaedic Surgery, University of California San Francisco, San Francisco, California, United States of America; Mayo Clinic Minnesota, UNITED STATES

## Abstract

Rotator cuff tears represent a large burden of muscle-tendon injuries in our aging population. While small tears can be repaired surgically with good outcomes, critical size tears are marked by muscle atrophy, fibrosis, and fatty infiltration, which can lead to failed repair, frequent re-injury, and chronic disability. Previous animal studies have indicated that Transforming Growth Factor-β (TGF-β) signaling may play an important role in the development of these muscle pathologies after injury. Here, we demonstrated that inhibition of TGF-β1 signaling with the small molecule inhibitor SB431542 in a mouse model of massive rotator cuff tear results in decreased fibrosis, fatty infiltration, and muscle weight loss. These observed phenotypic changes were accompanied by decreased fibrotic, adipogenic, and atrophy-related gene expression in the injured muscle of mice treated with SB431542. We further demonstrated that treatment with SB431542 reduces the number of fibro/adipogenic progenitor (FAP) cells—an important cellular origin of rotator cuff muscle fibrosis and fatty infiltration, in injured muscle by promoting apoptosis of FAPs. Together, these data indicate that the TGF-β pathway is a critical regulator of the degenerative muscle changes seen after massive rotator cuff tears. TGF-β promotes rotator cuff muscle fibrosis and fatty infiltration by preventing FAP apoptosis. TGF-β regulated FAP apoptosis may serve as an important target pathway in the future development of novel therapeutics to improve muscle outcomes following rotator cuff tear.

## Introduction

Rotator cuff (RC) tears are among the most common muscle-tendon injuries in our aging population, with an asymptomatic tear prevalence of 20% in patients aged 60–70 and a prevalence of greater than 50% in patients above 80 years old [1]. Approximately 250,000 RC repairs are performed by orthopaedic surgeons each year, representing a large annual healthcare expenditure, though still saving approximately $3.44 billion annually in lifetime societal savings compared to non-operative management [2].

While small cuff tears can be surgically repaired with good outcomes, medium-to-massive tears are often complicated by poor healing, failed repair, and frequent re-injury. Underlying muscle pathology, including atrophy and fatty infiltration, strongly contributes to the poor outcomes associated with larger tears [3,4], with atrophy and fatty infiltration identified as independent factors implicated in poor outcomes [5–7]. Muscle fibrosis is also consistently seen in animal injury models of RC tears [8–12], though its use as a clinical prognostic factor is limited by the inability of standard imaging techniques such as MRI to detect it in a clinical setting. Ultimately, there is no effective treatment to improve RC muscle quality after tendon tears at this time.

Despite an increasing body of new knowledge gained from many small and large animal RC injury models, the molecular and cellular mechanisms of rotator cuff muscle atrophy, fibrosis, and fatty infiltration remain largely undefined [8–12]. The Transforming Growth Factor-β (TGF-β) canonical signaling pathway is known to be important in the development pathologic fibrosis in multiple organ systems and tissues [13–15]. It has also been shown to be active in the setting of a combined tendon-nerve injury in rats, and to correlate with increased fibrosis and fatty infiltration of injured muscle [16]. Prior studies have likewise shown a correlation between p-SMAD2 activation and muscle fibrosis [13,15]. For these reasons, the canonical TGF-β pathway has been proposed as a master regulator of the fibrotic changes seen in muscle in chronic injury states.

Recent studies have identified a likely cellular source of fibrosis and fatty infiltration in dystrophic muscle. Fibro/adipogenic progenitor (FAP) cells, described by Joe et al [17] and Uezumi et al [18], have been shown to be a resident PDGFRα+ stem cell population within muscle with the potential to differentiate into fibroblasts and adipocytes. These cells appear to lack myogenic potential in their natural environment and have been shown to proliferate and differentiate in response to muscle injury [17–19]. Further work demonstrated that in muscular dystrophy (mdx) mice, the proliferation and survival of FAP cells depends on an intricate balance of TNF-α and TGF-β signaling, and that inhibition of TGF-β signaling through the use of the drug Nilotinib reduces FAP cell number and concurrent fibrosis in the mdx model [20].

In this study, we sought to test the feasibility of preventing rotator cuff muscle fatty infiltration and fibrosis using a small molecule TGF-β inhibitor SB431542 in a mouse model of RC tears. SB431542 is a potent inhibitor of activin receptor-like kinase (ALK)-4, ALK-5, and ALK-7 that has been shown to selectively block signaling through the TGF-β1 receptor [21]. By blocking downstream phosphorylation of SMAD2, SB431542 inhibits activin (via ALK-4) and TGF-β (via ALK-5) signaling without affecting BMP signaling, which occurs through phosphorylation of SMAD1 [21]. First identified by Callahan et al (2002; compound 14), SB431542 was noted to be the most potent ALK-5 inhibitor screened with an IC_50_ of 0.094μM, without inhibition of p38 kinase activity, and without any measurable cytotoxicity [22]. Although studies with SB431542 have not yet been conducted in humans, it has been used in numerous animal studies with no reported adverse effects.

We hypothesized that inhibition of TGF-β signaling with SB431542 would result in prevention of fibrosis and fatty infiltration after a massive RC tear, and that this process may be mediated by the impact of TGF-β signaling on FAP cell number.

## Methods

### Animal Surgeries

Adult C57B/6J female mice (N = 44) and female PDGFRα-GFP reporter mice (N = 6) underwent a complete supraspinatus and infraspinatus tendon transection (TT) and ipsalateral resection of a 5mm segment of the suprascapular nerve (DN). Sham surgery was performed on the contralateral side of each animal to serve as an internal control. All procedures were approved by the San Francisco Veterans Affairs Medical Center (SFVAMC) Institutional Animal Care and Use Committee (IACUC) (Protocol: 15–015). All animals were anesthetized with 1–5% isoflurane during surgery and received postoperative pain management with buprenorphine to minimize suffering according to our protocol. Of the 44 C57B/6J mice, 16 (N = 8/treatment group) were sacrificed at 2 weeks after injury for cell sorting analysis of FAP cells, 8 were sacrificed at 2 weeks for histological analysis (N = 4/group), and 20 were sacrificed at 6 weeks for histological and gene expression analysis (N = 4/group for histology; N = 6/group for gene expression analysis). The PDGFRα-GFP reporter mice were sacrificed at 2 weeks after injury for histological analysis (N = 3/group).

### SB431542 Administration

Beginning the day of surgery, mice were equally and randomly assigned to treatment or vehicle groups. One group underwent intraperitoneal (IP) injections with 10mg/kg SB431542 in 5% DMSO solution and the other group underwent IP injections with 5% DMSO solution (vehicle) daily for a total of 2 or 6 weeks. Animals were treated daily up until 24 hours prior to being sacrificed. Dosage was chosen based on the optimal inhibitory effect of 10mg/kg delivered intraperitoneally that was reported by previous studies [20, 23].

### Muscle Harvest

Animals were sacrificed at 2 and 6 weeks after surgery as detailed above. Supraspinatus muscles were harvested and weighed from all animals used for histological analysis (N = 4/timepoint/group for C57B/6J mice, 3/group for reporter mice) or gene expression analysis (N = 6/group, C57B/6J mice). Supraspinatus and infraspinatus were harvested and pooled for FACS (N = 8/group, C57B/6J mice).

### Histology

The muscles were snap frozen in liquid nitrogen-cooled isopentane and sectioned serially at -20°C at a thickness of 10μm with a cryostat. Masson trichrome staining was used to assess fibrosis. Oil red-O staining was used to assess fatty infiltration [8,10]. Fibrosis and fat indices were calculated for 4 whole muscle sections imaged at 5x from both surgical and sham sides per animal using the Adobe® Photoshop “Color Range” tool to quantify pixels corresponding to areas of muscle staining positive for fat or collagen as a fraction of cross-sectional muscle area, as previously described [24]. FAP cell count and apoptotic index calculations from PDGFRα-GFP reporter mice were performed using ImageJ (NIH) on four representative fields of view each from a different muscle section for each animal (N = 3/group).

### Apoptosis Assay

A fluorescent modified-TUNEL assay was performed on sections from PDGFRα-GFP reporter mice using ApopTag ® Red In Situ Apoptosis Detection Kit (EMD Millipore) following the manufacturer protocol. Sections were mounted on slides using VectaShield with DAPI and visualized using AxioVision software.

### Cell Sorting

Due to the small number of FAP cells in the atrophic RC muscle, we pooled the supraspinatus and infraspinatus muscles from 8 animals (in treated and vehicle groups, respectively) for FACS analysis. Cells were prepared for flow cytometry according to a protocol adapted from Joe, et al [17]. CD31-/CD45-/a7-/Sca1+ FAP cell population was sorted using the BD FACSAria™ II. Plots were generated with FlowJo V10.1.

### Real-Time Quantitative Reverse Transcription PCR

Total RNA was isolated using Trizol reagent (Invitrogen, Inc., Carlsbad, CA) according to the manufacturer's instructions. cDNA was synthesized using Transcriptor First Strand cDNA Synthesis Kit (Roche Applied Bioscience, Indianapolis, IN). qRT-PCR was performed to quantify the expression of the fibrotic markers PAI-1 and α-SMA and the adipogenic markers PPARγ and SREBP-1 in muscle samples using a SYBR Green I Master kit (Roche Applied Bioscience) with the primers found in S1 Table. Based on our previous study [25], four animals per group are needed to determine a significant difference in mTOR expression using the assumptions α = 0.05, β = 0.20, thus six biological replicates were used per group with three technical replicates each to account for any unanticipated variation. Gene expression was normalized to the housekeeping gene, 36B4. Fold change in mRNA expression was calculated by using ΔΔC_T_ as described previously [16,24].

### Statistical Analysis

For all analyses, a two-tailed Student’s T-test was used to assess for significance. Significance was defined as p<0.05. Data are presented as mean ± standard error of measurement.

## Results

### SB431542 reduced rotator cuff muscle fibrosis, fatty infiltration, and atrophy

**Fig 1:**
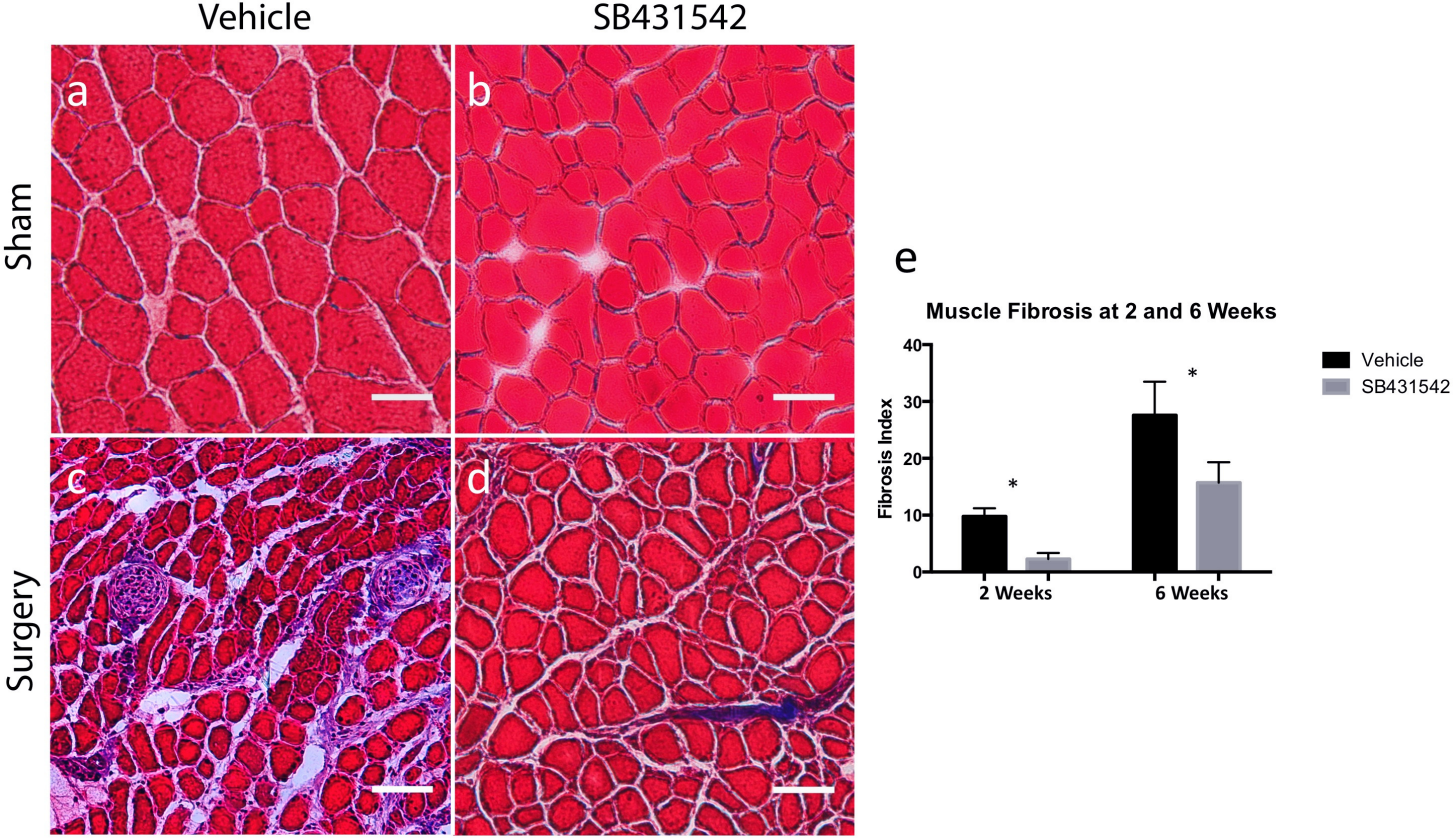
SB431542 reduces rotator cuff muscle fibrosis after injury. (a-d) Representative Masson trichrome stain of supraspinatus muscle sections at 6 weeks, scale bar = 50μm. (e) Quantification of injured muscle fibrosis indices at 2 and 6 weeks from 4 whole muscle sections/animal (N = 4/group) imaged at 5X magnification, *p<0.05.

At both 2 and 6 weeks after TT+DN injury, Masson trichrome staining revealed significantly decreased muscle fibrosis in the injured supraspinatus of mice that were treated with SB431542 compared to those that received vehicle (Fig 1A–1D), with a 6-week fibrosis index of 15.7 ± 3.6% in treated mice compared to 27.6 ± 5.9% in those that received vehicle (Fig 1E, p<0.05).

**Fig 2:**
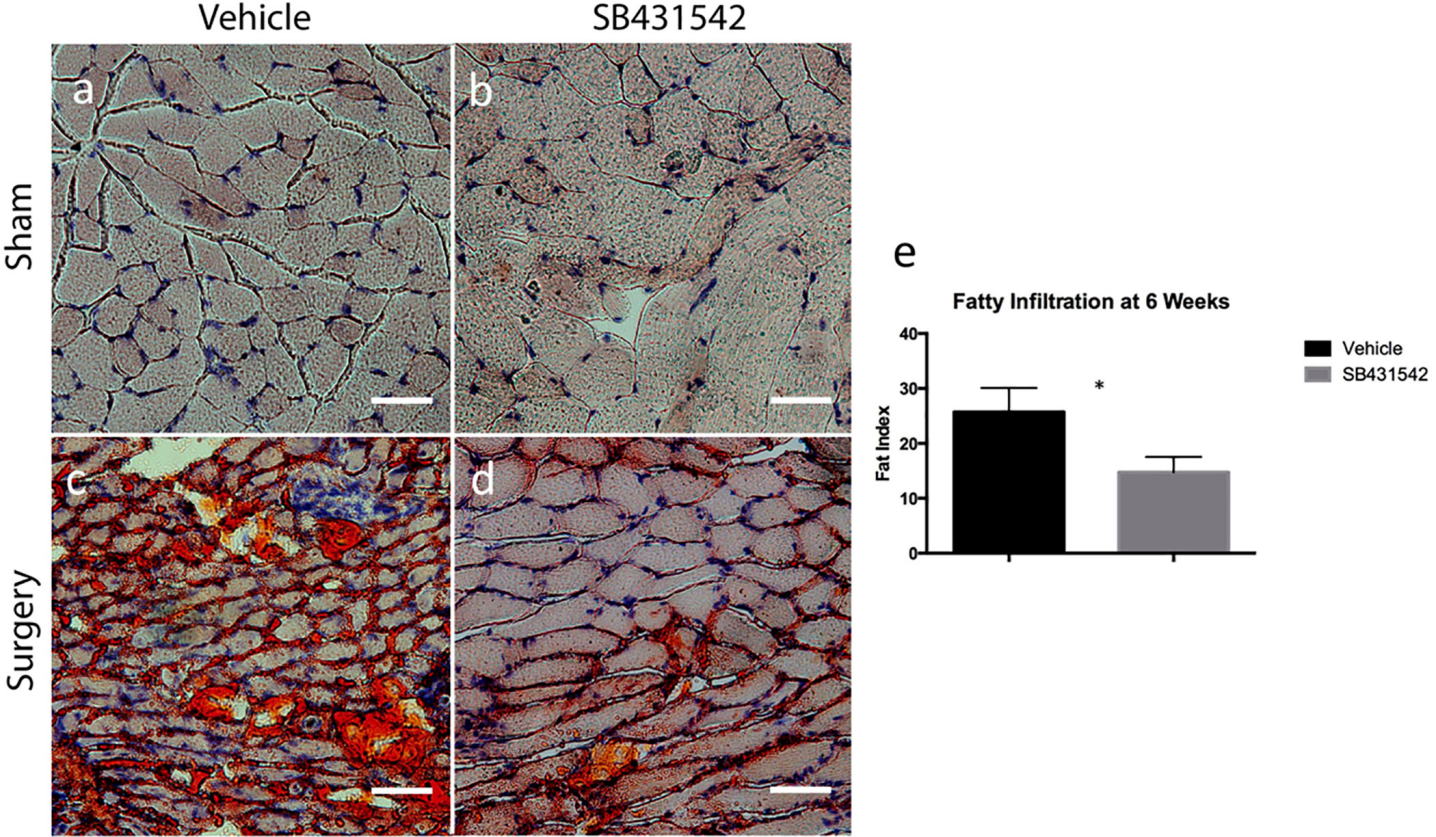
SB431542 reduces rotator cuff muscle fatty infiltration after injury. (a-d) Representative oil red-O stain of supraspinatus muscle sections at 6 weeks, scale bar = 50μm. (e) Quantification of injured muscle fat index indices at 6 weeks from 4 whole muscle sections/animal (N = 4/group) imaged at 5X magnification, *p<0.05.

As we have observed substantial fatty infiltration of injured muscle at 6 weeks in mice but not at timepoints as early as 2 weeks, oil red-O staining of muscle was done on mice treated at 6 weeks. Oil red-O revealed a decrease fatty infiltration in mice that received SB431542 compared to those that received vehicle (Fig 2A–2D), with a fat index of 14.8 ± 2.8% in treated mice compared to 25.7 ± 4.4% in those that received vehicle (Fig 2E, p<0.05).

**Fig 3:**
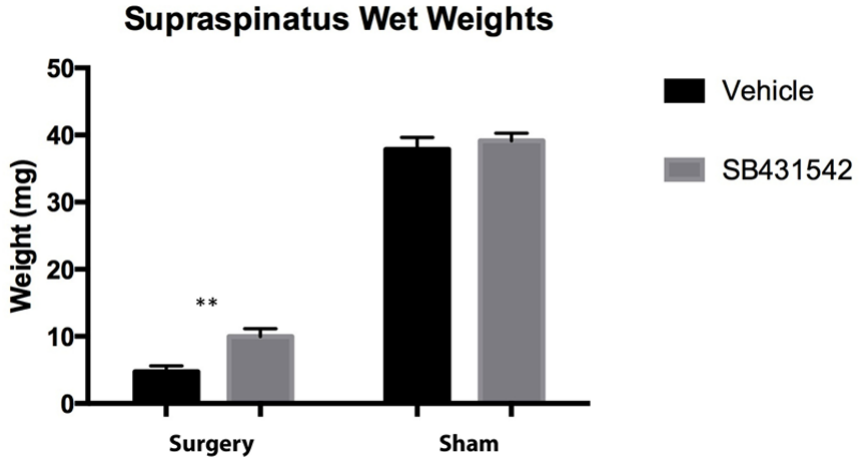
SB431542 decreases supraspinatus weight loss after injury. Graph shows average wet weight of freshly harvested supraspinatus muscles from both vehicle and treatment groups (N = 8/group). **p<0.01.

At 6 weeks after injury, injured supraspinatus muscles in mice that received vehicle showed an average wet weight of 4.7 ± 0.97 mg compared to an SB431542-treated weight of 10.0 ± 1.3 mg (Fig 3, p<0.01). There was no significant difference between the wet weights of sham-side muscles, with a weight of 37.9 ± 2.0 mg in the vehicle group compared to 39.2 ± 1.2 mg in mice that received treatment (Fig 3).

### SB431542 reduced fibrosis, fatty infiltration, and atrophy-related gene expression in injured rotator cuff muscle

**Fig 4:**
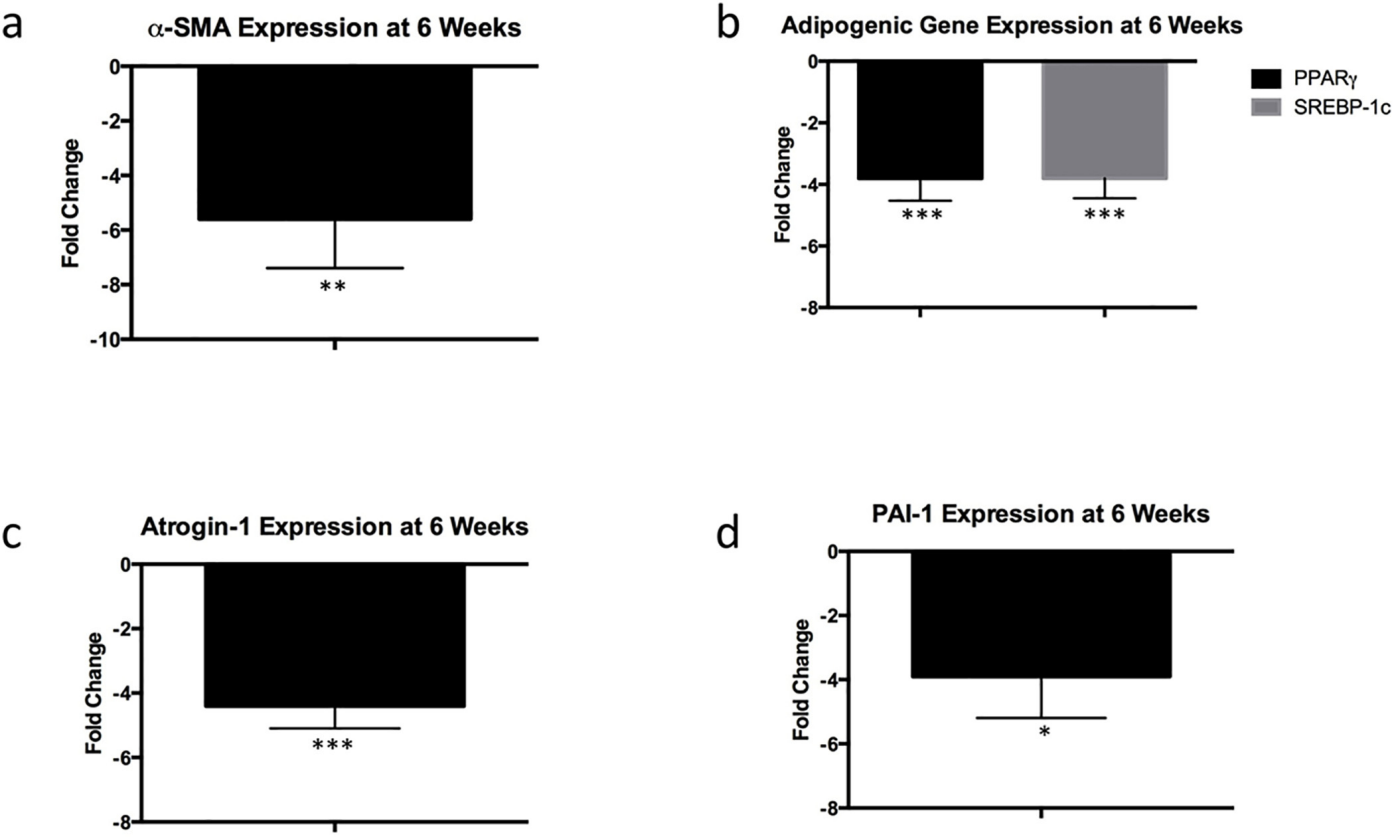
SB431542 decreases expression of fibrotic, adipogenic, and atrophy-related genes. (a) Fold change of α-SMA in injured muscle, SB431542 treatment compared to vehicle. (b) Fold changes of PPARγ and SREBP-1. (c) Fold change of atrogin-1. (d) Fold change of PAI-1. N = 6 animals/group, 3 technical replicates/animal. *p<0.05, **p<0.01, ***p<0.005.

At six weeks, a -5.6 ± 1.8 fold decrease was observed in the expression of α-SMA, a marker of myofibroblast activation, in the injured muscle of SB431542-treated mice compared to those that received vehicle (Fig 4A, p<0.01). The adipogenic transcription factors SREBP-1 and PPARγ were also downregulated, with fold changes of -3.8 ± 0.73 and -3.8 ± 0.72, respectively (Fig 4B, p<0.005 for both genes). The atrophy marker atrogin-1 was downregulated in injured muscle treated with SB431542 compared to control with a fold change of -4.4 ± 0.7 (Fig 4C, p<0.005). In treated mice, expression of the TGF-β1 target gene PAI-1 was decreased with a fold change of -3.9 ± 1.3 (Fig 4D, p<0.05). We did not observe any significant expression changes in sham-side muscle between the treatment and vehicle groups (S1 File).

### SB431542 reduced FAP cell number in rotator cuff muscle after injury

**Fig 5:**
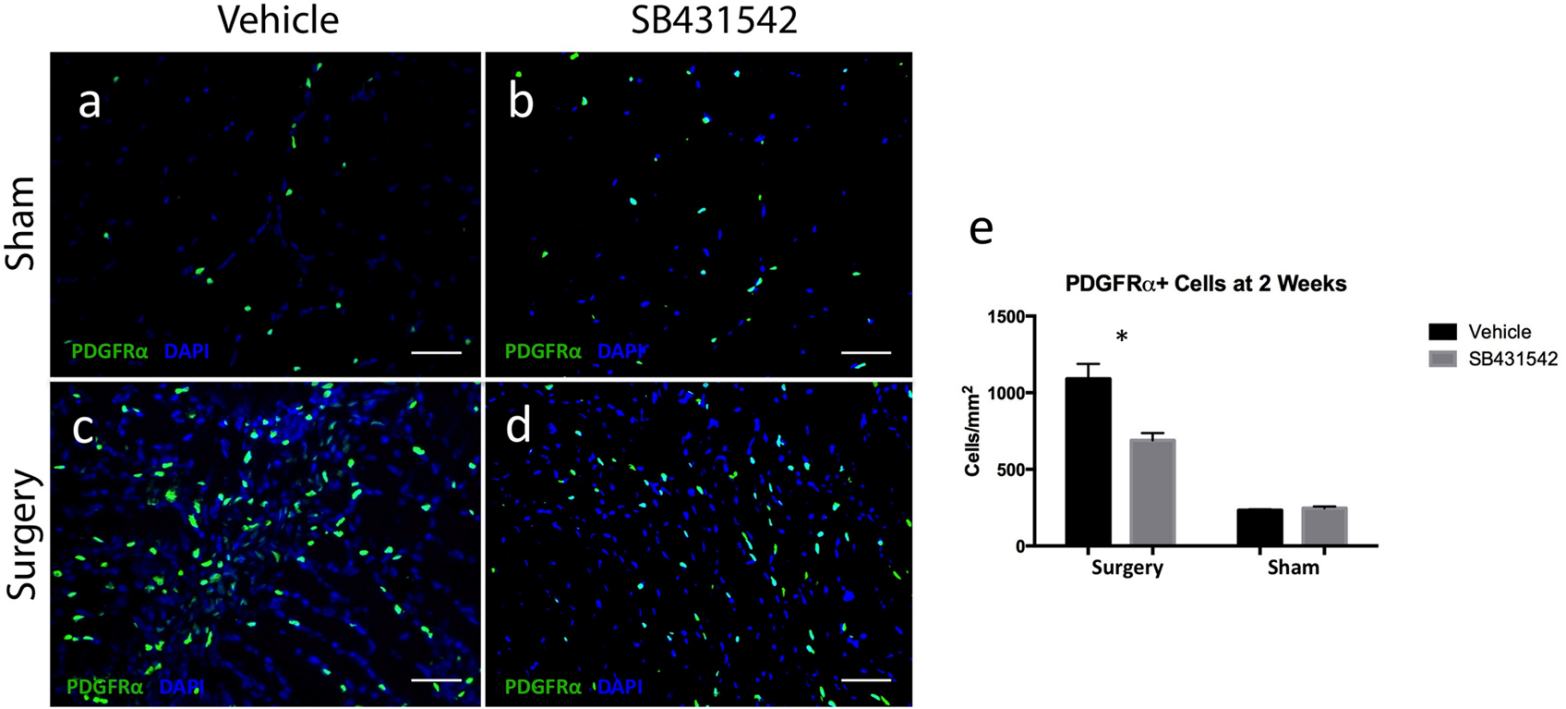
SB431542 reduces PDGFRα+ cell number in injured muscle. (a-d) representative supraspinatus muscle sections from PDGFRα-GFP reporter mice counterstained with DAPI, scale bar = 25 μm. (e) ImageJ quantification of PDGFRα+ cells/mm^2^ from 4 representative fields of view imaged at 20X magnification per animal (N = 3/group). *p<0.05.

Using PDGFRa-GFP reporter mice, we assessed FAP cell number in mice that had undergone TT+DN at two weeks after injury, and observed a substantial decrease in the number of PDGFRa + cells in injured muscle of mice that received treatment with SB431542, with approximately 689 ± 47 PDGFRα+ cells/mm^2^ in treated mice compared to 1091 ± 97 cells/mm^2^ in injured muscle of mice that received vehicle (p <0.05) (Fig 5C–5E). There was no significant difference in PDGFRα+ cell number in muscle from the sham sides of each group, with 247 ± 11 cells/mm^2^ in the treated group compared to 233 ± 7 cells/ mm^2^ in the group that received vehicle (5a-b, e).

**Fig 6:**
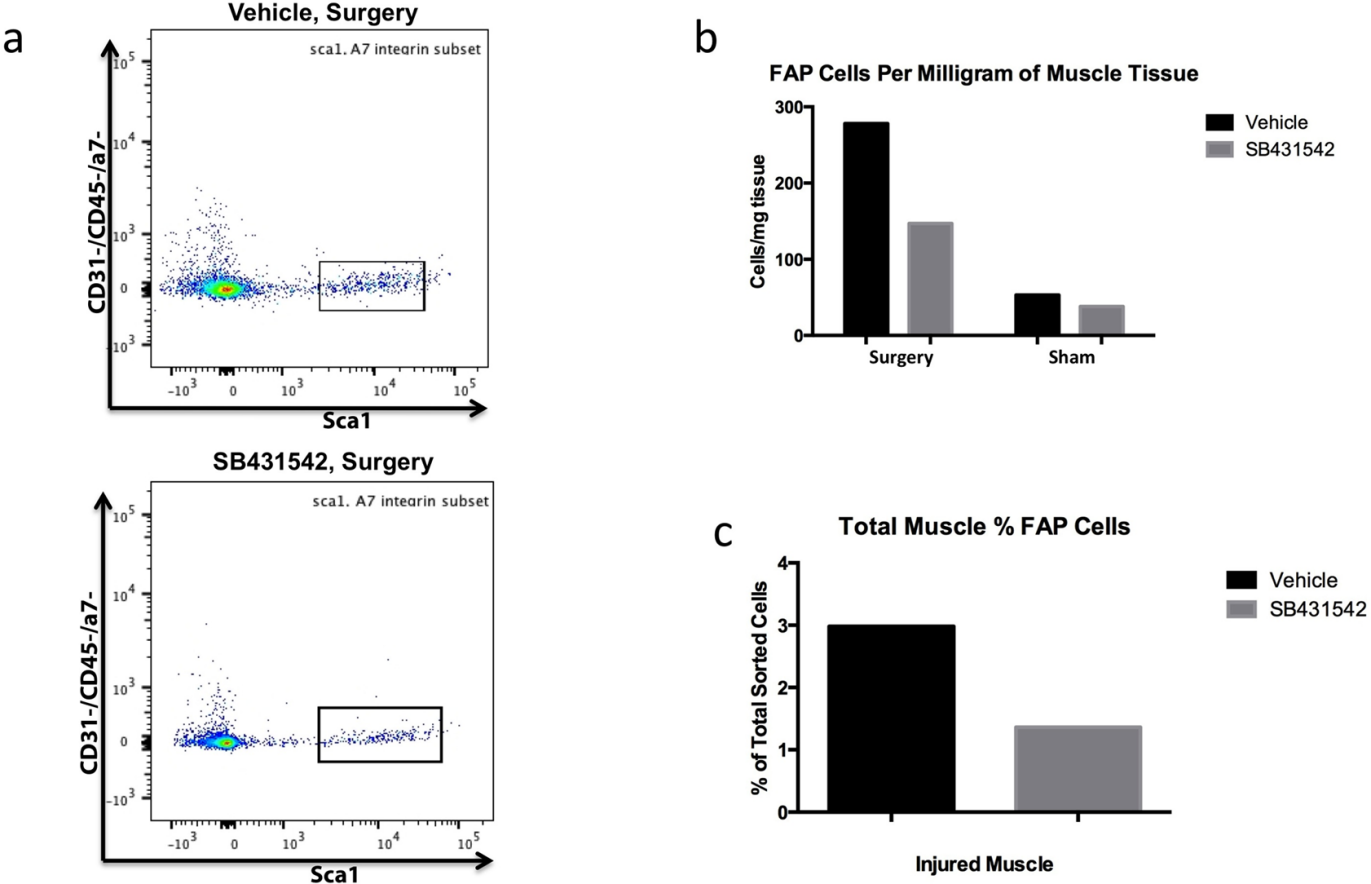
SB431542 decreases CD31-/CD45-/a7-/sca1+ FAP cell number at 2 weeks. (a) Representative flow cytometry overlay plots of cells sorted from vehicle group (upper plot) and SB431542 treatment group (lower plot); N = 8 supraspinatus, 8 infraspinatus pooled per group. Gate indicates sca1+, a7 integrin- FAP cells out of the CD31-/CD45- population. (b) Quantification of total CD31-/CD45-/a7-/sca1+ cell number per mg of sorted tissue. (c) Percent CD31-/CD45-/a7-/sca1+ cells of total cells sorted in injured muscle groups.

FACS analysis of FAP cell number performed on pooled supraspinatus and infraspinatus muscles from mice (N = 8/group) at 2 weeks after injury was supportive of the results seen on histology. SB431542 resulted in a decrease in CD31-/CD45-/a7-/sca1+ FAP cells in injured muscle, with 138 cells/mg muscle (1.36% of total cells sorted) in the treated group compared to 278 cells/mg (2.98%) in the group that received vehicle (Fig 6A–6C).

### SB431542 promotes FAP cell apoptosis in rotator cuff muscle after injury

**Fig 7:**
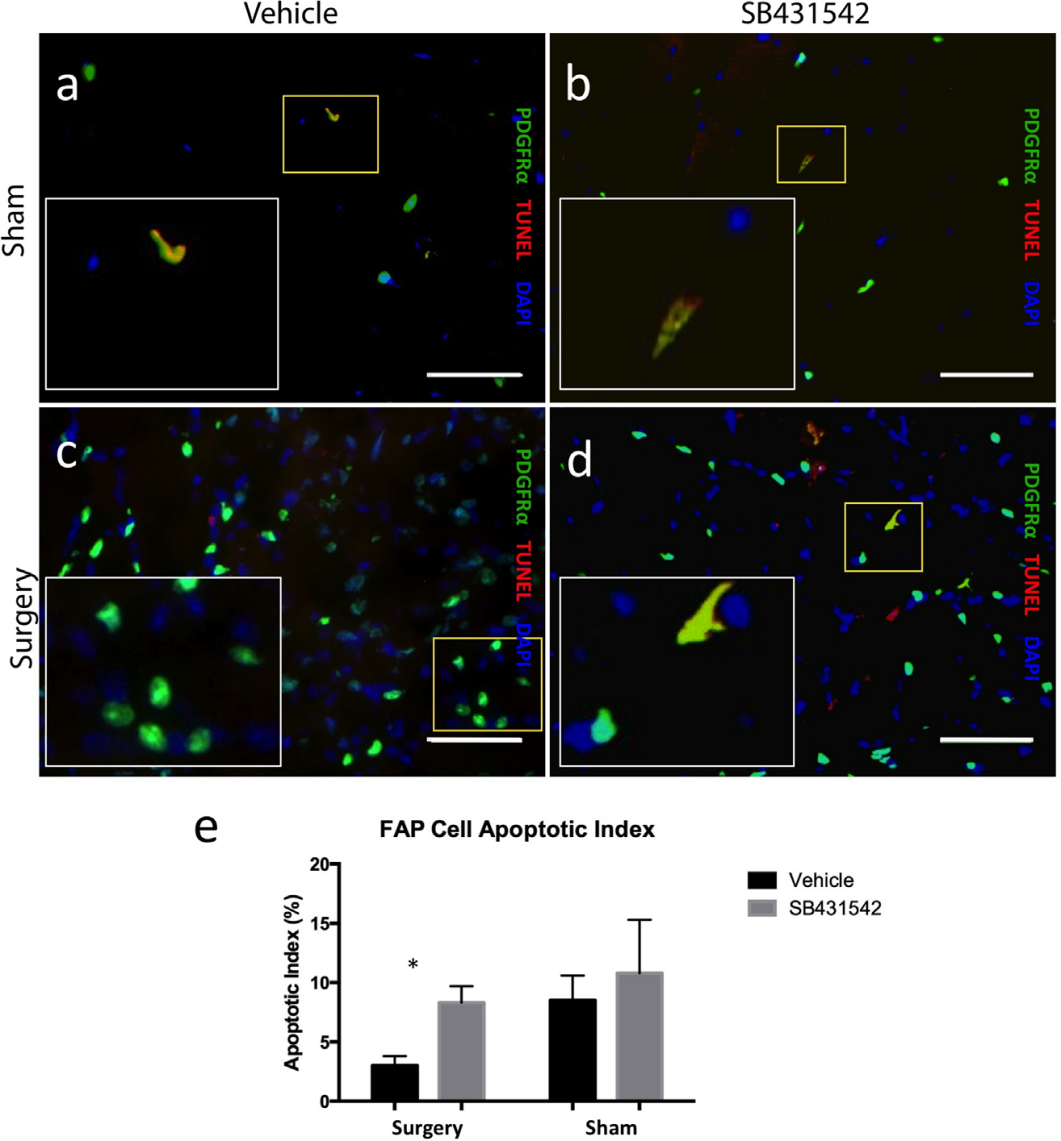
SB431542 promotes FAP cell apoptosis in injured rotator cuff muscle. (a-d) Representative fluorescent TUNEL assay of supraspinatus muscle from PDGFRα-GFP reporter mice, counterstained with DAPI, scale bar = 25 μm; selection in yellow box enlarged in right lower corner. (e) ImageJ quantification of TUNEL+ cells (rhodamine) that co-express PDGFRα (GFP) from 4 representative fields of view imaged at 20X magnification from PDGFRα-GFP reporter mice (N = 3/group). *p<0.05.

We performed a fluorescent modified-TUNEL assay on injured muscle from PDGFRα-GFP reporter mice (N = 3/group) to assess for apoptotic cells. We observed an increase in number of apoptotic cells in injured muscle of mice treated with SB431542 compared to vehicle (Fig 7C and 7D), with an FAP cell apoptotic index of 8.3 ± 1.4% in the injured muscle of mice treated with SB431542 compared to 3.0 ± 0.8% in the vehicle group (Fig 7E, p<0.05). There was no significant difference in apoptotic index of the sham-side muscle of both groups, with indices of 8.5 ± 2.1% and 10.9 ± 4.6% in the treatment and vehicle groups, respectively (Fig 7A and 7B).

## Discussion

Our previous work demonstrated significant up-regulation of TGF-β1 signaling in rotator cuff muscle after massive tendon tears, suggesting this pathway may play a critical role in rotator cuff muscle pathology [16]. In this study, we tested the feasibility of a small molecule TGF-β1 inhibitor, SB431542, in preventing rotator cuff atrophy, fibrosis and fatty infiltration in a preclinical mouse model of RC tear. Our results show that SB431542 inhibits TGF-β1 signaling and results in decreased muscle atrophy, fatty infiltration and fibrosis following a RC injury in a small animal model. Our data further suggest that this effect may be mediated by inducing apoptosis of a fibro/adipogenic progenitor cell population in RC muscles. Results from this study suggest that SB431542 may serve as a promising novel treatment for preventing further RC muscle degeneration in patients with critical size RC tears.

TGF-β describes a superfamily of polypeptide ligands which include TGF-β-like ligands (TGF-β1–3), activins, and the bone morphogenetic proteins (BMPs) that are expressed in a variety of tissues and play important roles in development, cellular proliferation and differentiation, and wound healing, among other processes [26–28]. TGF-β1 has been shown to be a secreted ligand from tissue macrophages, which converge at the site of injured tissues to help coordinate the repair process [20,29]. In the setting of sustained TGF-β signaling, a variety of organ systems and tissues, including the liver, lungs, and skeletal muscle, have been shown to develop a pathologic level of fibrosis [30–32]. TGF-β1 canonical pathway signaling occurs through heterodimerization of the TGF-β1 receptors I and II, which phosphorylate the cytoplasmic SMAD proteins, allowing them to act as transcription factors of many profibrotic targets [24–26]. SB431542 is a selective small molecule inhibitor of the activin receptor-like kinase 5 (ALK-5, also referred to as TGF-β1 Receptor I), with additional activity against ALK-4 and ALK-7, thus making it an effective inhibitor of the canonical TGF-β pathway by preventing the phosphorylation of SMAD2 [21,33]. It has been shown to not interfere with BMP signaling [21]. Previous studies with SB431542 have demonstrated its ability to block TGF-β1 signaling both in vitro and in vivo [34,35], though no trials in humans have yet been performed.

PAI-1 has been shown to respond sensitively and specifically to TGF-β1 signaling in a dose-dependent manner [36]. Besides serving as a TGF-β1 signaling activity indicator, PAI-1 has also been suggested to play a role in excessive accumulation of collagen in pathologic wound healing [37]. Studies have shown that the small molecule inhibitor, SB431542, is sufficient to reduce expression of PAI-1 [32], a finding which we have observed in our injury-treatment model (Fig 4D). Another marker of fibrosis that specifically corresponds to activated myofibroblasts is α-SMA [38]. In this study, SB431542 significantly reduced PAI-1 and α-SMA gene expression, resulting in reduced fibrosis in injured RC muscle. FAP cells are a proposed source of the α-SMA-expressing myofibroblasts seen in injured muscle tissue, and several studies have identified significant overlap in PDGFRα and TCF4 expression in fibro/adipogenic progenitors (also referred to as connective tissue fibroblasts in certain studies) and α-SMA expression [38–40]. Our results suggest that inhibition of TGF-β1 signaling in injured rotator cuff muscle results in a decrease in fibrogenic gene expression that correlates with less fibrosis on histology.

While other studies have examined the role of TGF-β1 signaling in the context of pathologic fibrosis, the specific role of TGF-β in adipogenesis and fatty infiltration following muscle injury remains largely unexplored. One reason for this may be that the rotator cuff appears to develop significantly more fatty infiltration after injury compared to other muscle groups such as the gastrocnemius [24]. In our study, we observe a substantial decrease in fatty infiltration of the injured rotator cuff following inhibition of TGF-β1 signaling that corresponds to a decrease in the expression levels of PPARγ and SREBP-1, two transcription factors that are known markers of adipogenesis within muscle [41–43]. The specific pathway through which TGF-β1 signaling leads to the upregulation of these markers in injured muscle remains unknown. Previously, it has been shown that inhibition of mTOR signaling through the use of the drug rapamycin leads to downregulation of PPARγ and a decrease in fat after TT+DN injury in rats [43]. Further studies are necessary to search for a direct link or crosstalk between the TGF-β1 and mTOR signaling pathways. However, this study suggests that inhibition of the TGF-β pathway is a potential method to decrease fatty infiltration after RC injury and repair.

TGF-β1 signaling has been linked to skeletal muscle atrophy through the activation of scleraxis and atrogin-1 [44]. Myostatin, a negative regulator of skeletal muscle size, has also been shown to signal through the ALK-4 and ALK-5 receptors [45], both of which are blocked by SB431542. A previous study demonstrated that SB431542 promotes growth of C2C12 myotubes in vitro and Xenopus muscle fibers ex vivo, though it may decrease the specific force of muscle fibers [46]. Here we show that, in vivo, SB431542 decreases the extent of muscle weight loss after a massive tendon-nerve injury in a manner that corresponds to a decrease in expression of atrogin-1 in the injured muscle. As SB431542 disrupts both myostatin and TGF-β1 signaling through the ALK-4/ALK-5 receptors, further studies are needed to determine the exact mechanism by which this improvement of muscle atrophy occurs.

The relationship of TGF-β1 signaling to the FAP cell response to muscle injury has been studied before in the context of mdx mice and muscle injury models; specifically, acute chemical injury (Lemos et al), repeated chemical injury, and sciatic nerve transection [20,47]. A review of these injury models demonstrates a marked contrast in the way that FAP cells respond to an acute versus a sustained muscle injury. In an acute chemical injury performed with BaCl_2_ injections into the tibialis anterior, FAP cells rapidly proliferate, reaching a peak number at about 3–4 days and returning to an uninjured baseline at roughly 9 days [20]. In this acute injury setting, investigators did not observe sustained activation of TGF-β1 signaling, in contrast to that seen in sustained injury models [20]. In sustained injury models such as sciatic nerve denervation that can be used to approximate chronic conditions, however, FAP cells maintain an increased concentration compared to uninjured muscle even at time points of 2 weeks or greater after the initial injury, and this elevated FAP cell number correlates with sustained TGF-β1 expression in the injured tissue [47]. Our previous study has suggested that FAPs are the important and major cellular source of fibroblasts and adipocytes in rotator cuff muscles after massive tendon tears [48]. In this study, we observed a significant reduction of FAP cell number accompanied by reduced muscle fibrosis and fatty infiltration in rotator cuff muscle after SB431542 treatment. These data further support our previous finding that FAPs are the major cellular source of rotator cuff muscle fibrosis and fatty infiltration.

Apoptosis, also known as programmed cell death, is an important mechanism regulating cell populations in many tissues. It is also an important regulator of the fate of stem cells. However, apoptosis of stem/progenitor cells in rotator cuff muscle pathology has not been intensively studied. Results from our current study suggest that TGF-β signaling may prevent FAP cells from undergoing apoptosis, resulting in a significantly increased FAP cell population, leading to fibrosis and fatty infiltration in rotator cuff muscle after a tendon-nerve injury. Importantly, we show that in uninjured (sham-side) muscle, FAP cells undergo apoptosis at a basal rate of ~9–11%, compared to a significantly lower rate of ~3% in untreated surgically injured muscle. This finding provides further evidence that increased TGF-β signaling, which is present in surgically injured but not sham muscle, is necessary to prevent this basal rate of apoptosis in FAP cells. Inhibiting TGF-β signaling with SB431542 counteracts this effect of TGF-β, thus promoting apoptosis of FAP cells, leading to reduction of the FAP cell population and reduced fibrosis and fatty infiltration in rotator cuff muscles. This finding further suggests that regulating FAP apoptosis is a worthwhile strategy in preventing muscle fibrosis and fatty infiltration.

The clinical implications of this study are highly relevant, not only because we have identified a potential therapeutic for a clinically relevant model, but because there are already pharmacologic agents on the market which have been shown to have the off-target effect of TGF-β1 inhibition. Such agents include the angiotensin II receptor blocker (ARB) losartan, which have already shown promise in the setting of treating cardiac and renal fibrosis [49, 50]. Future clinical studies may seek to evaluate the use of available pharmacologic inhibitors of TGF-β1 such as losartan in the setting of chronic muscle injuries in patients, given the promising results of inhibiting TGF-β1 signaling in murine tendon-nerve injuries.

One important clinical concern related to the use of TGF-β inhibition to address rotator cuff muscle degeneration, particularly in the immediate post-surgical setting, is the effect on tendon-to-bone healing. While TGF-β signaling may promote aberrant muscle fibrosis and fatty infiltration in the setting of a tendon-nerve injury, TGF-β1–3 isoforms may also play a role in tendon healing, particularly during the first couple of weeks after injury [51]. Of these isoforms, TGF-β3 signaling was shown to have the highest potency in stimulating COL1A1 and COL3A1 in rat cultured tendon fibroblasts, while TGF-β1 was found to counteract this fibrogenic activity in vitro, suggesting that TGF-β isoforms may have differing roles in tendon healing [51]. While studying the histological and biomechanical effects of SB431542 treatment on tendon healing was beyond the scope of this study, it would be important to establish the effect of TGF-β inhibition on these factors before considering treatment in humans.

A further clinical consideration is the role of TGF-β inhibition in the treatment of pre-existing muscle pathology as opposed to prevention of further muscle degeneration if treatment is initiated shortly after injury. In this study, we show the preventative effects of SB431542 treatment on muscle fibrosis and fatty infiltration when initiated immediately after a simulated massive rotator cuff tear. However, torn RC muscle in humans may undergo degeneration for months to years before a diagnosis is made. It will thus be important for future studies to consider the effects of TGF-β inhibition on previously injured muscle that has already developed significant pathology in order to more fully evaluate the clinical potential of this therapeutic approach.

An animal study of this type has certain inherent limitations. First, while the muscle pathology that we observe in mice closely mimics that seen in humans after RC tear, the mechanism of nerve injury in humans is thought to be a chronic process caused by increased traction on the suprascapular nerve that occurs gradually following tendon tear [52]. However, small animal models analogous to the one that we use have been validated by a number of other groups as closely mimicking the muscle pathology observed in humans [9, 53, 54]. Studies in larger mammals, including sheep, have also shown upregulation of the same adipogenic pathways that are active in this mouse model following injury [11]. Second, our method of quantifying histological changes is imperfect, as it relies on user selection of a defined pixel range and is subject to any artifact that may be introduced through the staining process itself. We therefore seek to minimize bias by analyzing four whole muscle sections per animal and by only comparing sections that were stained at the same time using the same protocol, thus controlling for any relative artifact introduced by the staining process. Future studies may involve the use of MRI for whole-muscle quantification of fatty infiltration, though this method would still be unable to accurately detect changes in muscle fibrosis. Additionally, we chose to study only one treatment dose of SB431542 based on previously reported inhibitory effects [20,23]. Given that we did not observe total prevention of fibrofatty infiltration following injury, it is possible that a higher dose of inhibitor might have proven more efficacious. We also chose to study only female mice, as we have not previously observed a difference in the response to TT+DN injury between male and female mice (unpublished data). However, future studies may randomize equal numbers of male and female mice to each treatment group to control for any unanticipated sex-related differences in the response to injury and treatment. Finally, as FAP cells are a small fraction of the cellular population within muscle, we chose to pool muscle from N = 8 mice per group for cell sorting analysis rather than run independent separate experiments. We cannot therefore perform a statistical analysis of this experiment, though the result was generated from a large number of animals and correlates closely with what we observe on histology and have quantified with ImageJ.

This study demonstrates the positive effects of TGF-β1 inhibition with SB431542 in preventing the outcomes of fibrosis and fatty infiltration in a mouse model of rotator cuff tear, and notes a strong correlation between these outcomes and the number of FAP cells present in the injured muscle at an earlier time point. We have provided further evidence to support the role of the canonical TGF-β signaling pathway as a master regulator of both the fibrotic and adipogenic changes in the setting of a clinically relevant chronic muscle injury and propose an exciting avenue of preventative treatment for muscle pathology following this exceedingly common muscle-tendon injury that currently presents a large burden to patients and the healthcare system.

## Supporting Information

S1 File Data Analysis. Quantification of fibrosis, fat, apoptotic indices, qRT-PCR expression analysis, PDGFRα+ cell count, muscle wet weights.
(XLSX)

S1 Table Primers used for Real-Time qRT-PCR. (PDF)
