# Supplementary material for: TGF-β Small Molecule Inhibitor SB431542 Reduces Rotator Cuff Muscle Fibrosis and Fatty Infiltration By Promoting Fibro/Adipogenic Progenitor Apoptosis
Source: PLoS One. 2016 May 17;11(5):e0155486. doi: 10.1371/journal.pone.0155486 (PMC4871364; doi:10.1371/journal.pone.0155486)
Supplement: S1 Table — (PDF) [file pone.0155486.s002.pdf]

Primers used for Real-Time qRT-PCR		
Gene	Forward (5' -> 3')	Reverse (5' -> 3')
36B4	GCGACCTGGAATCCAACTAC	ATCTGCTGCATCTGCTTGG
α-SMA	CAGGGAGTAATGGTTGGAAT	TCTCAAACATAATCTGGGTCA
PPARγ	CCACCAACTTCGGAATCAGCT	TTTGTGGATCCGGCAGTTAAGA
SREBP-1	AGCCGTGGTGAGAAGCGCAC	ACTGCTGCTGCCTCTGCTGC
Atrogin-1	GCAGAGAGTCGGCAAGTC	CAGGTCGGTGATCGTGAG
PAI-1	GGCCATTACTACGACATCCTG	GGTCATGTTGCCTTTCCAGT
